# Becoming Native-Like for Good or Ill: Online and Offline Processing of Case Forms in L2 Russian

**DOI:** 10.3389/fpsyg.2021.652463

**Published:** 2021-05-26

**Authors:** Natalia Cherepovskaia, Elizaveta Reutova, Natalia Slioussar

**Affiliations:** ^1^Department of Translation and Language Sciences, Universitat Pompeu Fabra, Barcelona, Spain; ^2^Department of Liberal Arts and Sciences, Saint Petersburg State University, Saint Petersburg, Russia; ^3^School of Linguistics, Higher School of Economics, Moscow, Russia

**Keywords:** second language acquisition, sentence processing, grammaticality illusion, syncretism, case, Russian

## Abstract

One of the central questions in second language processing studies is whether native (L1) and second language (L2) readers process sentences relying on the same mechanisms or there are qualitative differences. As their proficiency grows, L2 readers become more efficient, but it is difficult to determine whether they develop native-like mechanisms or rely on different strategies. Our study contributes to this debate by focusing on constructions that were demonstrated to cause characteristic *problems* in L1 processing: a particular type of case errors in Russian was taken as an example. We investigated how beginner and intermediate learners of Russian process such errors, measuring reading times and grammaticality judgment accuracy. At the beginner level, we found non-native-like patterns both in online and in offline measures. But at the intermediate level, native-like problems emerged in offline measures. In our view, this is a strong indication that these readers are using the same underlying mechanisms as in L1 processing. In online measures, L2 readers at both levels were, in general, much slower than native participants and exhibited characteristic non-native-like patterns, which we explained by delayed morphosyntactic processing. We conclude that our results are compatible with approaches, assuming that the mechanisms for L1 and advanced L2 processing are the same, but L2 processing is more cognitively demanding and therefore slower.

## Introduction

In studies of second language (L2) processing, the central question is whether the mechanisms and strategies it relies on are essentially the same as in the native language (L1) or there are qualitative differences. The answer to this question remains elusive: the obvious problems at the early stages of L2 acquisition might have different sources, and when performance subsequently improves and becomes more native-like, non-native-like strategies might underlie this achievement. In the present paper, we argue that focusing on constructions that were shown to cause characteristic processing *problems* for native speakers may help to shed new light on this question. Similar problems observed in L2 processing may be taken as an argument in favor of common underlying mechanisms – characteristic errors are definitely not one of the language learner’s goals, but a by-product of using a particular means to achieve a communicative goal.

Therefore, our aim was to select a processing problem characteristic of L1 readers and to investigate whether it can also be observed in L2 readers at different proficiency levels in online and offline measures. One type of processing problem that is extensively discussed in the experimental literature is grammaticality illusions. This term is used to describe the situation when a certain type of grammatical error is particularly difficult to detect. This is manifested in both online and offline measures: in reduced error-related reading time (RT) delays and in the higher proportions of incorrect answers in the speeded grammaticality judgment (GJ) task. In the present study, we selected a variety of case errors in Russian that were shown to give rise to grammaticality illusions (Slioussar and Cherepovskaia, 2014, 2021).

We conducted an experiment with three groups of L2 learners of Russian: an intermediate English-speaking group and two beginner groups (the speakers of English and the speakers of Spanish and Catalan). Participants read grammatical and ungrammatical sentences, including examples with different types of case errors, while RTs were measured and made GJs. Summarizing the results, we demonstrated that at the beginner level, both online and offline measures were influenced by factors that are not relevant to native speakers, but at the intermediate level, a native-like pattern emerged in offline measures. We argue that this happened when inflectional paradigms were acquired well enough – then L2 processing can rely on them in the same way as L1 processing does. However, intermediate L2 learners were still much less efficient than native speakers, which were reflected in the remaining differences in online measures. Finally, our study sheds new light on the underinvestigated topic of case processing by L2 readers.

The paper has the following structure. We start with a short overview of theoretical approaches to the differences in L1 and L2 processing. Then, we briefly present the relevant information on Russian grammar, on experimental studies of the Russian case system and grammaticality illusions in processing, before turning to our study.

### L1 and L2 Processing

Many authors assume that L1 and L2 processing mechanisms are qualitatively different but have divergent views on the source of these differences. According to the Shallow Structure Hypothesis (SSH; Clahsen and Felser, 2006a,b,c; Clahsen et al., 2010), L2 speakers are less sensitive to syntactic information in sentence processing and rely on semantic and pragmatic cues to a greater extent than L1 speakers. The Interface Hypothesis (IH; Sorace, 2011) suggests that near-native L2 speakers have difficulties with the integration of syntactic information and information from other cognitive domains.

The Bottleneck Hypothesis (Slabakova, 2009) claims that while L2 acquisition of semantics, syntax, and even pragmatics flow relatively smoothly, inflectional morphology is the major source of problems. These problems have a dramatic effect on processing because inflectional morphology encodes grammatical features and is the locus of crosslinguistic differences. Prévost and White (2000) proposed another morphology-based theory, the Missing Surface Inflection Hypothesis (MSIH), according to which the mapping of morphological forms to abstract grammatical categories is the weak link.

Another group of theories assumes that L1 and L2 might be different due to maturational changes in memory-processing mechanisms. For example, the Ullman’s declarative/procedural (DP) model (Ullman et al., 1997; Ullman, 2015, 2018) claims that learning abilities in the procedural memory peak during early childhood, while learning abilities in the declarative memory improve during childhood and early adulthood. Hence, L1 and L2 acquisition and processing rely on these two long-term memory systems to a different extent. In particular, procedural memory is responsible for generalized grammatical rules, which makes L1 processing faster and more automatic. Cunnings (2017) suggests that a primary source of L1/L2 processing differences lies in the ability to retrieve information from memory, and that L2 speakers are more susceptible to retrieval interference.

Now let us turn to the models assuming that L1 and L2 processing rely on the same mechanisms, and that the observed differences are due to independent factors. Firstly, L2 processing is cognitively more demanding (e.g., Hopp, 2006, 2010; McDonald, 2006), which might be due to lower automaticity and speed (Segalowitz, 2003; Segalowitz and Hulstijn, 2005; Jegerski, 2012; Kaan et al., 2015), limitations in lexical access (McDonald and Roussel, 2010), and syntactic integration (Hopp, 2014). Secondly, L2 processing may be less efficient due to interference from L1 (Sabourin and Haverkort, 2003; Hopp, 2006, 2010; McDonald, 2006; Basnight-Brown et al., 2007; Portin et al., 2007, 2008; Feldman et al., 2010; Jackson, 2010). Thirdly, L2 proficiency level plays a major role (Hopp, 2006; Gor and Jackson, 2013; Coughlin and Tremblay, 2015). For instance, Hopp (2006) showed that depending on their level, L2 readers process subject-object ambiguities more or less similarly to native speakers.

Cognitive resource limitations may also be responsible for the fact that L2 speakers perform better in offline experiments than in online ones (e.g., Hopp, 2010; López Prego and Gabriele, 2014). Interestingly, if the processing load increases in the online task, native speakers may demonstrate patterns similar to L2 learners. Based on this observation, Kaan et al. (2015) claims that L1 and L2 processing mechanisms are not different in nature, and the differences can be explained by the same factors that drive individual differences in L1 processing.

In the present paper, we aim to find out whether L2 readers gradually develop not only “good,” but also “bad” native-like processing patterns. The former may simply reflect their growing processing efficiency, while the latter is indicative of relying on the same processing mechanisms as those of the native speakers and may be used to tease apart the different theoretical approaches presented above.

### The Russian Case System

The Russian case system is complex, which makes it very difficult for L2 learners. Russian nouns are inflected for two numbers and six cases: nominative, genitive, dative, accusative, instrumental, and locative (also called as prepositional). The choice of case may be influenced by the syntactic role of the noun (subject, direct or indirect object, etc.), its semantic role (agent, patient, experiencer, etc.), and by the particular verb or preposition of this noun depends on. Moreover, the choice of inflection for a given case depends on the inflectional class and subclass the noun belongs to, which is determined based on several heterogeneous factors: the grammatical gender of the noun (masculine, feminine, or neuter), its animacy, and the phonological properties of the stem.

Adjectives and participles that modify nouns agree with them in number, case, and gender (only in the singular). They have separate sets of inflections. Table 1 provides two examples: the paradigms of the noun phrases *novyj stol* “new table_M_” and *novaja škola* “new school_F_.” Both nouns are inanimate and have the same non-palatalized consonant at the end of the stem, and the same adjective with a non-palatalized stem-final consonant is used in both phrases, so Table 1 does not illustrate the variation determined by these factors.

**Table 1:** Paradigms of the noun phrases *novyj stol* “new table_M_” and *novaja škola* “new school_F_.”

	Singular		Plural	
Nominative	*novyj stol*	*novaja škola*	*novye stoly*	*novye školy*
Genitive	*novogo stola*	*novoj školy*	*novyx stolov*	*novyx škol*
Dative	*novomu stolu*	*novoj škole*	*novym stolam*	*novym školam*
Accusative	*novyj stol*	*novuju školu*	*novye stoly*	*novye školy*
Instrumental	*novym stolom*	*novoj školoj*	*novymi stolami*	*novymi školami*
Locative	*novom stole*	*novoj škole*	*novyx stolax*	*novyx školax*

As Table 1 shows, the Russian case system involves complex patterns of syncretism. For example, adjective and noun inflections may coincide in nominative and accusative – this is true for all inanimate nouns in plural and for most inanimate and some animate nouns in singular. The genitive singular form of the noun *škola* “school_F_” is also syncretic with nominative and accusative plural, although the corresponding adjective forms are different; dative and locative singular have the same ending. As for adjectives, genitive, dative, instrumental, and locative forms coincide in feminine singular paradigms, and genitive and locative forms coincide in plural paradigms, although most corresponding noun forms are not syncretic. As we show below, syncretic adjective forms are crucial for the present study: they trigger the grammaticality illusions explored in our experiment.

Before we turn to grammaticality illusions, let us briefly review the previous studies of L2 processing of a case in Russian. While there is a substantial body of literature on L1 acquisition of the case system and several studies on L2 acquisition (e.g., Babyonyshev, 1993; Rubinstein, 1995a,b; Voeikova and Gagarina, 2002; Voeikova, 2011; Cherepovskaia et al., 2021), only a couple of papers are dedicated to L2 processing. They do not focus on case error processing, but their general conclusions are nevertheless relevant for our study.

Kempe and Mac Whinney (1998) compared English speakers learning Russian and German. In their experiment, participants were asked to perform a speeded picture choice task after hearing simple noun-verb-noun sentences. The influence of different factors was tested: word order (the canonical subject-first vs. the inverted object-first word order), animacy of the nouns, and case marking (only the nominative and accusative cases were investigated). The results demonstrated that the learners of Russian used case marking much more effectively than the learners of German. Kempe and MacWhinney concluded that this was because cases are a stronger cue in Russian, in spite of the complexity of the paradigm. Similar results were obtained in those authors’ following study (Kempe and Mac Whinney, 1999).

Gor et al. (2017) conducted two auditory lexical decision experiments that comparing native and non-native processing of different case forms. They used nominative and genitive forms with overt and zero inflections (as Table 1 shows, some Russian nouns have a zero inflection in the nominative singular and an overt inflection in the genitive plural, while for some other nouns, the opposite is true). Native speakers always processed nominative forms significantly faster than other forms, irrespective of the inflections (individual form frequency was taken into account). The performance of L2 learners who were native speakers of English depended on the task and on the proficiency level. In the first experiment, neither case nor inflection type significantly influenced reaction times. In the second experiment, more complex nonce stimuli were used: real stems combined with real inflections from a wrong paradigm, which made participants pay more attention to the morphological properties of the stimuli. As a result, a native-like pattern emerged in the more advanced L2 group. Gor et al. conclude that the main problem for non-native speakers is not the morphological decomposition, as some authors have suggested (e.g., Clahsen and Felser, 2006a), but recombining the information encoded in the stem and the affix.

In another auditory lexical decision study with cross-modal morphosyntactic priming, Gor et al. (2019) compared three cases: nominative, genitive, and instrumental. Adjectives agreeing with nouns served as primes. Native speakers demonstrated significant differences among all three cases, with nominative being the fastest and instrumental the slowest (as before, individual form frequencies were taken into account). This reflects the hierarchical structure of the nominal paradigm, where cases have different functional load and type frequency. Non-native participants (English speakers) were early (heritage) and late learners of Russian with different proficiency levels. For all of them, a significant difference between nominative and oblique cases was found, but highly proficient late learners showed a native-like difference between genitive and instrumental. This demonstrates the maturation of the case system, which we are also going to explore in the present study.

### Grammaticality Illusions

Grammaticality illusions are processing problems that have been studied in numerous experiments, predominantly with native speaker participants. Most studies have focused on grammaticality illusions in subject-verb agreement (also known as *agreement attraction*). In particular, they show that number agreement errors are more difficult to detect in sentences like (1a) than in sentences like (1b) (e.g., Clifton et al., 1999; Pearlmutter et al., 1999; Wagers et al., 2009; Dillon et al., 2013; Tanner et al., 2014). In other words, (1a) is likely to be erroneously perceived as grammatical – hence the term *grammaticality illusion*. This is manifested both in online and offline measures: in diminished error-related RT delays (e.g., Clifton et al., 1999; Pearlmutter et al., 1999; Wagers et al., 2009; Dillon et al., 2013), smaller P600 amplitudes in electroencephalographic studies (e.g., Tanner et al., 2014), and higher proportions of incorrect answers in GJ tasks (e.g., Wagers et al., 2009).

1.
  *The key to the cabinets were rusty.
  *The key to the cabinet were rusty.

There is general agreement that the grammaticality illusion in (1a) is triggered by the dependent noun: its plural feature disrupts the agreement between the subject noun and the verb, but different authors disagree about how exactly this happens. In their argumentation, they rely not only on processing, but also on production data: attraction errors are produced significantly more often than other agreement errors (e.g., Jespersen, 1924; Quirk et al., 1972; Bock and Miller, 1991; Vigliocco et al., 1995, 1996; Franck et al., 2002, 2006; Hartsuiker et al., 2003; Solomon and Pearlmutter, 2004; Eberhard et al., 2005; Staub, 2009, 2010). Existing approaches can be divided into two groups: some assume that the number representation on the noun phrase is faulty or ambiguous, while others argue that attraction takes place when we try to retrieve the agreement controller.

Agreement attraction has been studied not only in English, but also in many other languages. In Russian, it has been observed in number, gender, and person agreement (Nicol and Wilson, 1999; Yanovich and Fedorova, 2006; Lorimor et al., 2008; Laurinavichyute and Vasishth, 2016; Slioussar and Malko, 2016; Slioussar, 2018). A number of studies investigated subject-verb agreement violations and attraction in L2 (e.g., Nicol and Greth, 2003; Hoshino et al., 2010; Lim and Christianson, 2015; Jegerski, 2016; Lago and Felser, 2018). While non-native speakers may be less sensitive to some factors like animacy or the conceptual number of the noun (as opposed to the grammatical number), they show native-like agreement attraction patterns. This can be explained by the fact that the phenomenon relies on very general mechanisms in production and comprehension and is found across languages. Therefore, for our study, we selected a different type of grammaticality illusion that relies on particular features of Russian grammar.

Consider the examples in (2a–c; 2a) is grammatical, while in (2b) and (2c), the noun *gorod* “town” is in the wrong case. The form of the adjective modifying this noun is syncretic, and this was demonstrated to trigger grammaticality illusions in sentences like (2b) (Slioussar and Cherepovskaia, 2014, 2021). These errors cause shorter RT delays and higher proportions of incorrect answers in the speeded GJ task than other case errors, like the one in (2c). This happens despite the fact that the preposition *o* “about” can be used only with locative, which should resolve the ambiguity of the adjective form and predetermine the case of the noun. Syncretic adjective forms not only disrupt error detection in comprehension, but also increase error rates in production: Rusakova (2013) demonstrated this for naturally occurring errors, and Slioussar et al. (2017) for errors occurring in experimental conditions.

1.
  - *Knigi o russkix gorod**ax** byli interesnymi.*
    book_NOM.PL_ about Russian_LOC.PL(=GEN.PL)_ town_LOC.PL_ were interesting
    ‘The books about Russian towns were interesting.’
*Knigi o russkix gorod**ov** byli interesnymi.
    book_NOM.PL_ about Russian_LOC.PL(=GEN.PL)_ town_GEN.PL_ were interesting
*Knigi o russkix gorod**am** byli interesnymi.
    book_NOM.PL_ about Russian_LOC.PL(=GEN.PL)_ town_DAT.PL_ were interesting

Slioussar and Cherepovskaia (2014, 2021) showed that grammaticality illusions can be observed with prepositions requiring different cases and with different syncretic adjective forms. There are different approaches to syncretism in theoretical morphology (e.g., Zwicky, 1991; Blevins, 1995; Stump, 2001; Bobaljik, 2002; Baerman et al., 2005; Müller, 2011), relying on the underspecification of inflectional morphemes, referral rules, etc. Grammaticality illusions discussed in this paper do not allow teasing them apart (although see some speculations in Slioussar and Cherepovskaia, 2021) – they only prove that syncretism is somehow represented in the mental lexicon.

As for the particular mechanisms underlying these illusions, Slioussar and Cherepovskaia (2014, 2021) suggested the following explanation, relying on their data and on other processing studies dealing with syncretism as well as on the retrieval approach to subject-verb agreement attraction (e.g., Solomon and Pearlmutter, 2004; Lewis and Vasishth, 2005; Badecker and Kuminiak, 2007; Wagers et al., 2009; Dillon et al., 2013): native speakers can predict the case of a noun based on the preposition, so the system detects a mismatch in sentences like (2b) and (2c). The violation of expectations always triggers rechecking: in particular, in (2b) and (2c) the parser tries to find out where the unexpected genitive or dative case came from. Syncretic forms activate not only the relevant set of features, but also – to a lesser extent – all sets for which they are ambiguous; so in examples like (2b), the system may retrieve the genitive plural feature set from the syncretic adjective form, which may lead to the wrong conclusion that the sentence is grammatical, i.e., to a grammaticality illusion.

### The Present Study

The goal of the present study was to find out whether grammaticality illusions described in the previous section for L1 processing can also be found in L2 processing. As we noted above, for these illusions to be possible, syncretism should be somehow represented in the mental grammar (existing studies do not favor a particular theoretical approach to syncretism). We hypothesize that if L2 learners develop the relevant representations at all, this happens only when the system matures, i.e., not at the beginner level, but at more advanced proficiency levels. If this causes L2 learners to develop a processing pattern that is analogous to that of native speakers’ – i.e., specific problems with detecting particular case errors – this may be used as an argument in favor of similar L1 and L2 processing mechanisms.

For our study, we recruited three groups of L2 learners of Russian: two beginner groups with different native languages (English and Catalan and Spanish) and one upper-intermediate group of English native speakers. A control group of native speakers also participated in the study. We collected online and offline data using self-paced reading to measure word-by-word RTs and GJ.

Foreshadowing the results, we can say that RT patterns were similar in the three L2 groups and different from those of native speakers: for all L2 readers, genitive plural forms were especially difficult, while for L1 readers, no case form was more difficult than the others. The distribution of errors in GJs in the upper-intermediate group resembled those of native speakers, while the two beginner groups showed a different pattern. These results support the approaches arguing for similar processing mechanisms in L1 and L2, but indicate that for these mechanisms to start working, the representation of L2 grammar should reach a certain level. A non-native-like pattern in online measures points to the role of morphological complexity in L2 processing that plays no role in L1 processing (genitive plural has the largest variety of inflectional affixes in the plural subparadigm).

## Experiment

### Participants

Three groups of learners of Russian volunteered to participate in the experiment. Group 1 (*English-speaking upper-intermediates*) included 29 native speakers of American English (15 females), aged 20–26 (mean age 23.7). They were students at different American universities; at the time of the experiment, they were participating in an exchange program with Saint Petersburg State University in Russia. To enter the program, an upper-intermediate proficiency level (B2) in Russian was required. The students took part in the experiment after spending approximately 2 months in Russia.

Group 2 (*Spanish-Catalan-speaking beginners*) included 33 Spanish-Catalan bilinguals (19 females), aged 20–38 (mean age 25.6). They were studying Russian at the University of Barcelona and at the A. Pushkin Institute of Russian Language in Barcelona. They had passed their A1 level exams approximately 8 months before participating in the experiment and had approximately 1 month to study before their A2 level exam. At the time of the experiment, they had never been to Russia either to study the language or as tourists.

Group 3 (*English-speaking beginners*) included 51 native speakers of American English (34 females), aged 20–28 (mean age 24.3). They were studying Russian at different universities in the USA. They had passed their A1 level exams approximately 6–8 months before participating in the experiment and had approximately 1–3 months to study before their A2 level exam. Like the participants from Group 2, they had never been to Russia.

Finally, a control group of native Russian speakers was recruited in Saint Petersburg. This group included 36 participants (20 females), aged 20–25 (mean age 22.5).

The experiment was carried out in accordance with the Declaration of Helsinki and existing Russian and international regulations concerning ethics in research. All participants provided informed consent.

Upper-intermediate English-speaking participants and Spanish-Catalan-speaking beginners were recruited from two particular language-learning programs and tested one by one in a quiet room. English-speaking beginners were recruited from Russian learning programs at several American universities and tested online. Thus, this group was potentially less homogeneous, but the fact that the results were very similar in the two beginner groups shows that the observed pattern was not accidental, and the differences between these groups and the upper-intermediate group cannot be associated either with different native languages or with different experimental settings.

### Materials

We constructed 27 sets of target sentences. Every set consisted of a grammatically correct sentence and two versions with case errors. All sentences contained six words and had the same syntactic structure: a subject noun in nominative plural modified by a prepositional phrase (a preposition, an adjective, and a target noun) and a predicate (the verb *byli* “were” and an adjective or a participle). We selected prepositions that require locative, genitive, or dative case. An example of a genitive preposition set is given in (3a–c); an example of a locative preposition set is presented in (2a–c) above. Target nouns could appear in genitive, locative, and dative plural – depending on the preposition, one case form was correct and two others were ungrammatical.

1.
  - *Fil’my bez izvestnyx akter**ov** byli skučnymi.*
    movie_NOM.PL_ without famous_GEN.PL(=LOC.PL)_ actor_GEN.PL_ were boring
    ‘The movies without famous actors were boring.’
*Fil’my bez izvestnyx akter**ax** byli skučnymi.
    movie_NOM.PL_ without famous_GEN.PL(=LOC.PL)_ actor_LOC.PL_ were boring
*Fil’my bez izvestnyx akter**am** byli skučnymi.
    movie_NOM.PL_ without famous_GEN.PL(=LOC.PL)_ actor_DAT.PL_ were boring

As we discussed in the introduction, the syncretism of adjective forms in the genitive and locative plural triggers grammaticality illusions in native speakers: errors like (3b) are less noticeable than other case errors, as in (3c; Slioussar and Cherepovskaia, 2014, 2021). We will call them *target* and *control errors*. The resulting experimental conditions are listed in Table 2.

**Table 2:** Nine experimental conditions.

	Prepositions taking locative: nine sets	Prepositions taking genitive: nine sets	Prepositions taking dative: nine sets
Target nouns in locative	*CR: L-L*: correct form, as in (2а)	*TE: G-L*: target error, as in (3b)	*CE: D-L*: control error, as in (4c)
Target nouns in genitive	*TE: L-G*: target error, as in (2b)	*CR: G-G*: correct form, as in (3а)	*CE: D-G*: control error, as in (4b)
Target nouns in dative	*CE: L-D*: control error, as in (2c)	*CE: G-D*: control error, as in (3c)	*CR: D-D*: correct form, as in (4а)

Thus, we had locative and genitive preposition sets with target and control errors. We used the following abbreviations for the experimental conditions: for example, *CR: L-L* (a grammatically correct sentence: a preposition taking the locative case with a target noun in locative), *TE: L-G* (a sentence with a target error: a preposition taking the locative case with a target noun in genitive), *CE: L-D* (a sentence with a control error: a preposition taking the locative case with a target noun in the dative). Following Slioussar and Cherepovskaia (2014, 2021), we used dative forms as control errors^1^ and added dative preposition sets, as in (4a–c), to our materials.

1.
  - *Učitelja po inostrannym jazyk**am** byli xorošimi.*
    teacher_NOM.PL_ on foreign_DAT.PL_ language_DAT.PL_ were good
    ‘The teachers of foreign languages were good.’
*Učitelja po inostrannymjazyk**ax** byli xorošimi.
    teacher_NOM.PL_ on foreign_DAT.PL_ language_LOC.PL_ were good
*Učitelja po inostrannym jazyk**ov** byli xorošimi.
    teacher_NOM.PL_ on foreign_DAT.PL_ language_GEN.PL_ were good

Dative plural adjective forms are not morphologically ambiguous, so these sets contain no target errors. They were used to balance the stimuli (so that genitive, locative, and dative target nouns were equally frequent as correct forms and as errors) and to compare different case errors in a situation, where no grammaticality illusions are expected. For native speakers, Slioussar and Cherepovskaia (2014, 2021) found no difference between the *CE: D-L* and *CE: D-G* conditions, either in RTs or in GJ results. This confirmed their conclusion that the differences observed in the locative and genitive preposition sets were indeed due to grammaticality illusions, and other factors did not play a significant role.

Otherwise, our materials were different from those of Slioussar and Cherepovskaia (2014, 2021). We simplified the syntactic structure of target sentences and tried to select only the high frequent words that would be familiar to learners of Russian from very early on. To do so, we relied on several textbooks of Russian as a foreign language that were used at the universities our participants attended (e.g., Nummikoski, 1996; Lubensky et al., 2001; Kagan et al., 2005; Lekić et al., 2008). All target word forms were 6–9 letters long.

In total, 62 sentences were included: 27 target sentences (nine grammatical and 18 ungrammatical) and 35 fillers (22 grammatical and 13 ungrammatical, with subject-predicate agreement errors to make the task more diverse). We distributed target sentences among three experimental lists using the Latin square principle. As a result, each list contained one sentence from every target set. Fillers were the same in every list. During the experiment, participants were assigned to one of the three lists and presented with target and filler sentences from their list in a random order.

### Procedure

For Groups 1 and 2 and for native speakers, the experiment was run on a PC using Presentation software.^2^ For Group 3, it was run on a web-based platform using Ibex Farm (Drummond, 2013). This method was found to be reliable in several previous psycholinguistic studies including those dedicated to L2 processing (e.g., Lago et al., 2019).

We used the word-by-word self-paced reading methodology (Just et al., 1982). Each trial began with a screen presenting a sentence, in which the words were masked by dashes, while spaces and punctuation remained intact. Each time the participant pressed the space bar, a word was revealed, the previous word was re-masked, and RTs were measured.

At the end of each sentence, participants were asked whether the sentence they had read was grammatically correct and gave a yes/no response by button press. Participants were instructed to read at a natural pace and to give their responses as quickly as possible. Four practice items were presented before the beginning of the experiment.

Thus, we combined self-paced reading and GJ tasks in one experiment, while Slioussar and Cherepovskaia (2014, 2021) used them separately, as is customary in L1 studies. In Slioussar and Cherepovskaia’s self-paced reading experiments, no more than one-sixth of stimulus and filler sentences contained errors, and comprehension questions rather than grammaticality questions were used so as not to attract readers’ attention to errors and not to disrupt their natural reading patterns. In another experiment, Slioussar and Cherepovskaia used the speeded GJ method, because a non-speeded task would be too simple for native speakers.

With L2 readers, the situation is different. Even our upper-intermediate group made a lot of errors in the non-speeded GJ task. As for RT patterns, we ran an additional pilot experiment with a group of 10 upper-intermediate English-speaking students who did not take part in the main study. We used the same stimulus sentences and added grammatically correct filler sentences with the same syntactic structure (with prepositions requiring genitive, locative, or dative case, as in the main study), so that only one-quarter of the sentences contained errors. Instead of GJ questions, we asked comprehension questions with a choice of two answers. This pilot experiment revealed the same tendency that we found in the main study: genitive plural forms were more difficult to process than locative and dative ones. This confirmed the validity of our decision to collect online and offline data from L2 participants in one study.^3^

We recruited a control group of native speakers using the same experimental design, but, as could be expected, the task was too easy for them. They made virtually no GJ errors. Unlike the L2 participants, their RT patterns changed compared to the self-paced reading experiments of Slioussar and Cherepovskaia (2014, 2021), which did not focus the readers’ attention on errors. Therefore, below we will compare our L2 groups both to the control native speaker group and to the results reported by Slioussar and Cherepovskaia, since the complexity of their tasks is more appropriate for L1 readers.

### Analysis

We analyzed participants’ RTs and GJ accuracy. Only items for which the grammaticality question was answered correctly were included in the RT analysis. Every target sentence contained six words, or regions, for which RTs were measured. RTs that exceeded a threshold of 2.5 standard deviations, by region and by condition, were excluded (Ratcliff, 1993). In total, 2.4% of the data was excluded in the NS Group, 3.2% of the data in Group 1, 4.7% of the data in Group 2, and 9.5% of the data in Group 3.

The statistical analysis was done in the *R* programming environment.^4^ We modeled RT data with a mixed-effects regression using the *lmer* function from the *lme4* package, and GJ data with a mixed-effects logistic regression using the *glmer* function from the *lme4* package (Bates et al., 2015). To obtain the values of *p* from the *t* values given by the model, we used the *lmerTest* package (Kuznetsova et al., 2015). For *post hoc* analyses, Tukey’s tests were conducted using the *glht* function from the *multcomp* package (Bretz et al., 2010). Random intercepts and random slopes by a participant and by an item were included in the models.

We started by analyzing sentences from the locative, genitive, and dative preposition sets separately in every group. As we showed in Table 2, in every set the target noun could be used in three different cases (one grammatically correct condition and two conditions with errors). We used mixed-effects regressions to estimate the differences between conditions in every region, treating the case of the target noun as a factor of interest. The correct case was taken as the reference level. Then, when two conditions with errors were compared, dative was taken as the reference level in the locative and genitive sets, and genitive in the dative sets.

We noticed that L2 readers processed genitive plural target nouns slower than dative and locative ones, independently of any other factors. To estimate this statistically, we used mixed-effects regressions on all data from the region containing the target noun in every group. We treated the case of the target noun as a factor of interest. First, the dative case was taken as the reference level and then genitive to compare the two remaining cases.

As for GJs, we analyzed sentences from the locative, genitive, and dative preposition sets using a mixed-effects logistic regression. The case on the target noun was the factor of interest. As with RTs, the correct case was taken as the reference level. Then, when two conditions with errors were compared, dative was taken as the reference level in the locative and genitive sets, and genitive in the dative set. After looking at every group separately, we analyzed the three L2 groups together.

### Results and Discussion

#### Control Group: Native Speakers of Russian

##### Reaction Times

**Figure 1:**
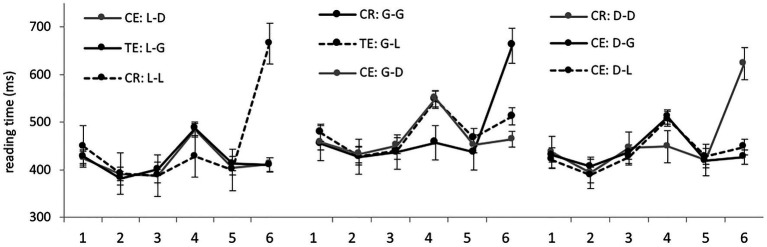
Native speaker group: average RTs per region (in ms) in the nine experimental conditions.

Average RTs per region in different experimental conditions are presented in Figure 1. Let us first discuss the results obtained for the locative, genitive, and dative preposition sets separately. The results with *p* < 0.05 are reported as statistically significant (for all such results, model outputs are presented in Table 3). In all sets, there were no significant differences in regions 1–3 before the target noun, as expected: these regions contain the same words in different conditions.

**Table 3:** Model outputs for RT analyzes in different conditions.

Group	Set	Comparisons	Region	Model outputs
NS	Locative	*CR: L-L* vs. *TE: L-G*	4	*β* = −65.59, *SE* = 21.13, *t* = −3.10, *p* < 0.01
NS	Locative	*CR: L-L* vs. *CE: L-D*	4	*β* = −64.36, *SE* = 21.34, *t* = −3.02, *p* < 0.01
NS	Locative	*CR: L-L* vs. *TE: L-G*	6	*β* = 266.34, *SE* = 36.45, *t* = 7.31, *p* < 0.01
NS	Locative	*CR: L-L* vs. *CE: L-D*	6	*β* = 266.47, *SE* = 36.55, *t* = 7.29, *p* < 0.01
NS	Genitive	*CR: G-G* vs. *CE: G-D*	4	*β* = −91.33, *SE* = 24.26, *t* = −3.77, *p* < 0.01
NS	Genitive	*CR: G-G* vs. *TE: G-L*	4	*β* = −92.44, *SE* = 24.33, *t* = −3.80, *p* < 0.01
NS	Genitive	*CR: G-G* vs. *CE: G-D*	6	*β* = 195.79, *SE* = 28.78, *t* = 6.80, *p* < 0.01
NS	Genitive	*CR: G-G* vs. *TE: G-L*	6	*β* = 167.07, *SE* = 28.95, *t* = 5.77, *p* < 0.01
NS	Dative	*CR: D-D* vs. *CE: D-G*	4	*β* = −62.76, *SE* = 18.70, *t* = −3.36, *p* < 0.01
NS	Dative	*CR: D-D* vs. *CE: D-G*	4	*β* = −52.06, *SE* = 18.59, *t* = −2.80, *p* = 0.01
NS	Dative	*CR: D-D* vs. *CE: D-G*	6	*β* = 196.87, *SE* = 25.22, *t* = 7.81, *p* < 0.01
NS	Dative	*CR: D-D* vs. *CE: D-G*	6	*β* = 178.41, *SE* = 25.30, *t* = 7.05, *p* < 0.01
Gr1	Locative	*CR: L-L* vs. *TE: L-G*	4	*β* = −1371.2, *SE* = 214.1, *t* = −6.41, *p* < 0.01
Gr1	Locative	*CE: L-D* vs. *TE: L-G*	4	*β* = 1039.5, *SE* = 211.4, *t* = 4.92, *p* < 0.01
Gr1	Locative	*CR: L-L* vs. *TE: L-G*	5	*β* = 384.02, *SE* = 75.87, *t* = 5.06, *p* < 0.01
Gr1	Locative	*CR: L-L* vs. *CE: L-D*	5	*β* = 577.96, *SE* = 71.05, *t* = 8.14, *p* < 0.01
Gr1	Locative	*CR: L-L* vs. *TE: L-G*	6	*β* = 1497.9, *SE* = 189.9, *t* = 7.88, *p* < 0.01
Gr1	Locative	*CR: L-L* vs. *CE: L-D*	6	*β* = 1744.5, *SE* = 177.6, *t* = 9.82, *p* < 0.01
Gr1	Genitive	*CR: G-G* vs. *CE: G-D*	5	*β* = 289.66, *SE* = 67.03, *t* = 4.32, *p* < 0.01
Gr1	Genitive	*CR: G-G* vs. *TE: G-L*	5	*β* = 274.44, *SE* = 81.19, *t* = 3.38, *p* < 0.01
Gr1	Genitive	*CR: G-G* vs. *CE: G-D*	6	*β* = 1549.0, *SE* = 192.5, *t* = 8.28, *p* < 0.0
Gr1	Genitive	*CR: G-G* vs. *TE: G-L*	6	*β* = 1178.2, *SE* = 234.6, *t* = 5.02, *p* < 0.01
Gr1	Dative	*CR: D-D* vs. *CE: D-G*	4	*β* = 851.4, *SE* = 221.1, *t* = 3.85, *p* < 0.01
Gr1	Dative	*CR: D-D* vs. *CE: D-G*	5	*β* = −224.29, *SE* = 83.02, *t* = −2.83, *p* = 0.01
Gr1	Dative	*CR: D-D* vs. *CE: D-L*	5	*β* = −252.86, *SE* = 84.58, *t* = −2.99, *p* < 0.01
Gr1	Dative	*CR: D-D* vs. *CE: D-G*	6	*β* = −802.0, *SE* = 125.9, *t* = −6.37, *p* < 0.01
Gr1	Dative	*CR: D-D* vs. *CE: D-L*	6	*β* = −995.4, *SE* = 127.2, *t* = −7.83, *p* < 0.01
Gr1	All sets	Gen vs. Loc	4	*β* = −477.14, *SE* = 184.89, *t* = 2.58, *p* = 0.02
Gr1	All sets	Gen vs. Dat	4	*β* = 488.23, *SE* = 173.24, *t* = 2.82, *p* = 0.01
Gr2	Locative	*CR: L-L* vs. *TE: L-G*	4	*β* = −1816.5, *SE* = 310.2, *t* = −5.86, *p* < 0.01
Gr2	Locative	*CR: L-L* vs. *CE: L-D*	4	*β* = −700.9, *SE* = 328.3, *t* = −2.16, *p* < 0.01
Gr2	Locative	*CE: L-D* vs. *TE: L-G*	4	*β* = −1115.6, *SE* = 352.6, *t* = 3.16, *p* < 0.01
Gr2	Locative	*CR: L-L* vs. *TE: L-G*	6	*β* = 946.5, *SE* = 313.7, *t* = 3.02, *p* < 0.01
Gr2	Locative	*CR: L-L* vs. *CE: L-D*	6	*β* = 844.8, *SE* = 313.5, *t* = 2.70, *p* = 0.01
Gr2	Genitive	*CR: G-G* vs. *TE: G-L*	6	*β* = 1164.31, *SE* = 385.10, *t* = 3.02, *p* < 0.01
Gr2	Genitive	*CR: G-G* vs. *CE: G-D*	6	*β* = 1224.87, *SE* = 340.42, *t* = 3.60, *p* < 0.01
Gr2	Dative	*CR: D-D* vs. *CE: D-G*	4	*β* = −1378.3, *SE* = 280.7, *t* = −4.91, *p* < 0.01
Gr2	Dative	*CE: D-G* vs. *CE: D-L*	4	*β* = 949.9, *SE* = 284.3, *t* = 3.34, *p* < 0.01
Gr2	Dative	*CR: D-D* vs. *CE: D-G*	6	*β* = 741.0, *SE* = 261.5, *t* = 2.83, *p* = 0.01
Gr2	Dative	*CR: D-D* vs. *CE: D-L*	6	*β* = 766.0, *SE* = 271.8, *t* = 2.82, *p* = 0.01
Gr2	All sets	Gen vs. Loc	4	*β* = 1044.68, *SE* = 171.12, *t* = 6.11, *p* < 0.01
Gr2	All sets	Gen vs. Dat	4	*β* = 945.73, *SE* = 172.82, *t* = 5.47, *p* < 0.01
Gr3	Locative	*CR: L-L* vs. *TE: L-G*	4	*β* = −2349.1, *SE* = 554.0, *t* = −3.43, *p* < 0.01
Gr3	Locative	*CR: L-L* vs. *CE: L-D*	4	*β* = 1879.8, *SE* = 457.5, *t* = 3.12, *p* < 0.01
Gr3	Genitive	*CR: G-G* vs. *CE: G-D*	6	*β* = 1748.8, *SE* = 381.0, *t* = 2.89, *p* < 0.01
Gr3	Dative	*CR: D-D* vs. *CE: D-G*	4	*β* = 2105.4, *SE* = 521.1, *t* = 3.25, *p* < 0.01
Gr3	Dative	*CR: D-D* vs. *CE: D-G*	6	*β* = −1708.8, *SE* = 398.5, *t* = −3.08, *p* < 0.01
Gr3	Dative	*CR: D-D* vs. *CE: D-L*	6	*β* = −1336.4, *SE* = 375.6, *t* = −2.77, *p* = 0.02
Gr3	All sets	Gen vs. Loc	4	*β* = 704.35, *SE* = 287.51, *t* = 2.45, *p* = 0.04
Gr3	All sets	Gen vs. Dat	4	*β* = 752.48, *SE* = 295.86, *t* = 2.52, *p* = 0.04

In region 4 (the target noun), correct case forms were processed significantly faster than incorrect forms in all sets. There were no differences between various errors. Thus, when L1 readers focus on error detection, grammaticality illusions disappear. No differences reached significance in region 5, while in the final region, region 6, grammatically correct sentences were processed significantly slower than incorrect ones in all sets. Presumably, in the latter case, the readers already knew the answer to the grammaticality question after detecting an error, while in the former, they spent some time rechecking that there were no errors.

We also looked at the processing times of target nouns depending on their case, taking data from all sets together. However, average RTs hardly differed: 484 ms for genitive plural and 493 ms for locative and dative plural. Accordingly, the analysis yielded no significant results.

##### Grammaticality Judgments

L1 readers made only three GJ errors, which constituted less than 0.01% of answers and were clearly accidental.

#### Group 1: English-Speaking Upper-Intermediates

##### Reaction Times

**Figure 2:**
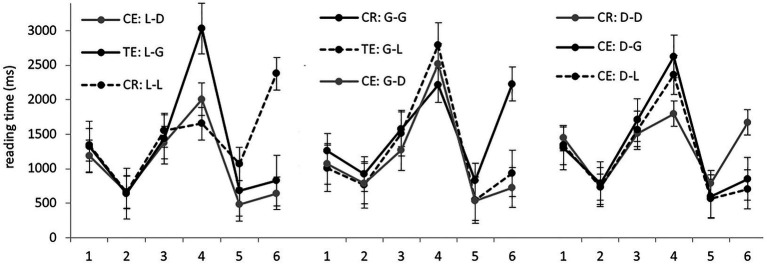
Group 1: average RTs per region (in ms) in the nine experimental conditions.

The average RTs per region in different experimental conditions are presented in Figure 2. Analyzing data from the locative, genitive, and dative preposition sets separately, we found significant differences only in regions 4–6 (model outputs are presented in Table 3). In regions 5 and 6 after the target noun, RTs in the correct conditions were significantly longer than in the ungrammatical ones. This result is similar to the L1 group: if a case error was detected in region 4, the remaining words could be read faster because participants already knew the answer to the grammaticality question.

Now let us focus on region 4, containing the target noun. In the locative sets, genitive forms were read significantly *slower* than other forms, grammatical or ungrammatical. So the pattern was different both from the L1 control group and from the results obtained by Slioussar and Cherepovskaia (2014, 2021): in the former case, different case errors were processed equally slowly, while in the latter, genitive errors were processed *faster* than dative ones due to a grammaticality illusion. In the genitive sets, there were no significant differences in region 4. Thus, we found no evidence of grammaticality illusions; moreover, correct case forms were not processed significantly faster than incorrect ones. In the dative sets, genitive errors took significantly longer than correct forms, while the difference between the latter and locative errors did not reach significance.

Then we analyzed all the data from region 4 together. Genitive forms were processed significantly slower (2,626 ms on average) than both locative (2,269 ms) and dative (2,110 ms). No significant differences between locative and dative forms were found.

Let us summarize the results in region 4. In the native speaker group and in the experiments of Slioussar and Cherepovskaia (2014, 2021), the difference between grammatical and ungrammatical case forms was significant in all sets, while case marking *per se* was not a significant factor. Slioussar and Cherepovskaia (2014, 2021) also observed grammaticality illusion effects. In Group 1, as well as in the two other L2 groups to be discussed below, the grammaticality factor did not always reach significance, and there was no evidence of grammaticality illusions. But case marking affected RTs.

This result cannot be explained by case frequency or the order of acquisition. Genitive is much more frequent in the Russian language than locative and dative.^5^ L2 learners acquire genitive later than locative, but earlier than dative (see Rubinstein, 1995a,b; Cherepovskaia et al., 2021). However, this result can be explained by morphological complexity. Many inflectional classes and subclasses that have different case affixes in singular, use the same affixes in the plural, but genitive plural is an exception (this is partly illustrated in Table 1). Four affixes with different orthographic variants are used in genitive plural; the choice between them is regulated by relatively complex rules and depends on the inflectional class, the last consonant of the stem, and some other factors. We will come back to this question in more detail in the General Discussion section.

##### Grammaticality Judgments

The numbers and percentages of incorrect responses in different experimental conditions are presented in Table 4. The resulting picture is very similar to that observed in native speakers: conditions with target errors (where grammaticality illusions are expected) triggered more incorrect answers than conditions with control errors. In the sentences with prepositions requiring locative case, genitive errors were significantly more difficult to detect than dative errors (*β* = 1.61, *SE* = 0.48, *z* = 3.37, *p* < 0.01).^6^ In the genitive sets, there was an even more pronounced difference between locative and dative errors (*β* = 2.26, *SE* = 0.41, *z* = 5.51, *p* < 0.01). Furthermore, the grammaticality illusion condition (with locative errors) was significantly different from the correct condition (*β* = 1.82, *SE* = 0.38, *z* = 4.74, *p* < 0.01), while the condition with control dative errors was not. In the dative sets, where no grammaticality illusions were expected because adjective forms are morphologically unambiguous, there were no significant differences.

**Table 4:** Incorrect answers in different conditions.

Condition	Required case	Used case	Incorrect answers		
			Group 1	Group 2	Group 3
CR: L-L	Loc	Loc	15 (17%)	33 (33%)	48 (31%)
TE: L-G	Loc	Gen	25 (29%)	46 (46%)	54 (35%)
CE: L-D	Loc	Dat	9 (10%)	47 (47%)	52 (34%)
CR: G-G	Gen	Gen	21 (24%)	37 (37%)	49 (32%)
TE: G-L	Gen	Loc	51 (59%)	50 (51%)	63 (41%)
CE: G-D	Gen	Dat	15 (17%)	43 (43%)	56 (37%)
CR: D-D	Dat	Dat	7 (8%)	33 (33%)	51 (33%)
CE: D-G	Dat	Gen	14 (16%)	43 (43%)	54 (35%)
CE: D-L	Dat	Loc	15 (17%)	49 (49%)	61 (40%)
Total			172 (22%)	381 (43%)	488 (35%)

#### Group 2: Spanish-Catalan-Speaking Beginners

##### Reaction Times

**Figure 3:**
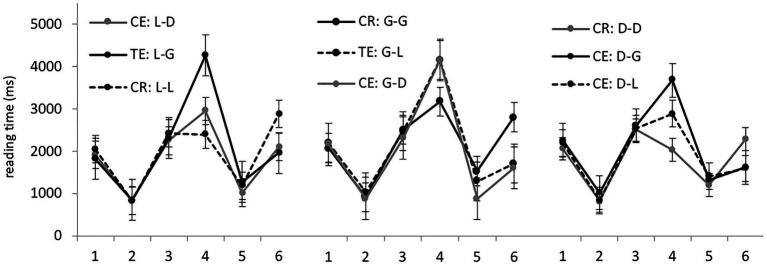
Group 2: average RTs per region (in ms) in the nine experimental conditions.

Average RTs per region in different experimental conditions are presented in Figure 3. We started by analyzing sentences from the locative, genitive, and dative preposition sets separately. Significant differences were found only in region 4, containing the target noun, and in the sentence-final region, region 6 (model outputs are presented in Table 3). In region 6, correct conditions were processed significantly slower than conditions with errors in all three sets.

As in Group 1, region 4 showed no evidence of grammaticality illusions. In the locative sets, all three conditions differed significantly, with the correct locative forms being processed the fastest and genitive forms the slowest. In the genitive sets, there were no significant differences in this region. In the dative sets, correct noun forms differed significantly from genitive forms, but not from locative forms; genitive forms also took significantly longer than locative forms.

Analyzing all the data from region 4, containing the target noun, we found the same pattern as in Group 1. Genitive forms were read significantly slower (3,796 ms on average) than locative (3,231 ms) and dative ones (3,114 ms). The difference between genitive and the two other cases was significant.

##### Grammaticality Judgments

The numbers and percentages of incorrect responses in different experimental conditions are presented in Table 4. First of all, it is evident that the experimental task was difficult for beginner learners: on average, 43% of answers were incorrect, while the upper-intermediate Group 1 gave only 22% incorrect answers. Secondly, Group 1 demonstrated a native-like pattern, while Group 2 was non-native-like both in online and in offline measures. Target errors did not differ significantly from control errors, and, in fact, no differences between experimental conditions reached significance: apparently, all target sentences, both grammatical and ungrammatical, were difficult to judge for the beginner L2 readers.

#### Group 3: English-Speaking Beginners

##### Reaction Times

**Figure 4:**
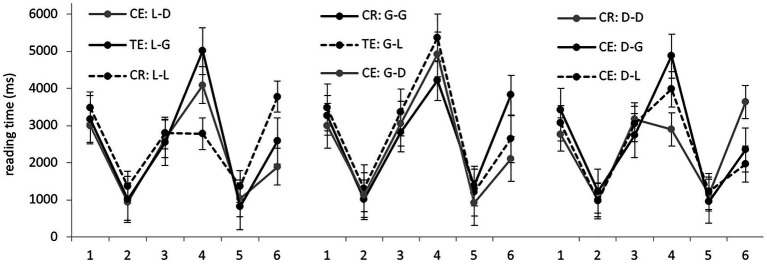
Group 3: average RTs per region (in ms) in the nine experimental conditions.

Average RTs per region in different experimental conditions are presented in Figure 4. We started by analyzing sentences from the locative, genitive, and dative preposition sets separately. Significant differences were found only in region 4, containing the target noun, and in the sentence-final region, region 6 (model outputs are presented in Table 3). As before, there was no evidence of grammaticality illusions. In region 6, grammatical sentences took significantly longer than ungrammatical ones with dative forms in the locative and genitive sets. In the dative sets, grammatical sentences were significantly different from both ungrammatical conditions.

Now let us look at target nouns in region 4. In the locative sets, correct noun forms were significantly different from genitive forms, but not from dative forms. In the genitive sets, there were no significant differences. In the dative sets, only the difference between the grammatical dative and ungrammatical genitive forms was significant.

Analyzing the data from the all sets together, we found the same picture as in Groups 1 and 2. Genitive forms were processed significantly slower (4,714 ms on average) than both locative (4,054 ms) and dative ones (3,973 ms). The latter two were not significantly different.

##### Grammaticality Judgments

The numbers and percentages of incorrect responses in different experimental conditions are presented in Table 4. We do not observe a native-like pattern in the upper-intermediate Group 1; as in the Spanish–Catalan-speaking beginner group, no differences between experimental conditions reached significance. This confirms the conclusion we reached earlier: all target sentences were difficult by those judge at the beginner level.

#### All L2 Groups

##### Reaction Times

Analyzing the three groups separately, we could preliminarily conclude that in online measures, they showed the same non-native-like pattern. In region 4, containing the target noun, no grammaticality illusions were found; the differences between grammatical and ungrammatical forms did not always reach significance, but genitive forms were processed slower than locative and dative ones. Therefore, analyzing data from all L2 groups together, we ran a mixed-effects regression on RTs from region 4, treating the group and the case of the target noun as factors of interest. Dative case and Group 3 (as the most numerous group) were taken as reference levels. For all statistically significant results, model outputs are given in Table 5.

**Table 5:** All L2 groups: model outputs for RT and GJ analyzes.

Statistical test	Factors/comparisons	Model outputs
Mixed-effects regression on RTs from region 4 (Dat and Gr3 are taken as reference levels)	Gen	*β* = 877.87, *SE* = 74.80, *t* = 11.74, *p* < 0.01
	Gr1	*β* = −1850.12, *SE* = 212.41, *t* = −8.71, *p* < 0.01
	Gr2	*β* = −1063.73, *SE* = 211.18, *t* = −5.04, *p* < 0.01
	Gen: Gr1	*β* = −595.86, *SE* = 136.79, *t* = −4.36, *p* < 0.01
Tukey contrasts on RTs from region 4	Gr1 vs. Gr2	*β* = −963.1, *SE* = 222.6, *t* = −4.33, *p* < 0.01
	Gr1 vs. Gr3	*β* = −2087.6, *SE* = 200.1, *t* = −10.43, *p* < 0.01
	Gr2 vs. Gr3	*β* = −1124.5, *SE* = 193.7, *t* = −5.81, *p* < 0.01
	Gen vs. Loc	*β* = 658.16, *SE* = 57.43, *t* = 11.46, *p* < 0.01
	Gen vs. Dat	*β* = 749.87, *SE* = 56.67, *t* = 13.23, *p* < 0.01
Mixed-effects regression on GJs (control and Gr3 are taken as reference levels)	Gr1	*β* = −1.28, *SE* = 0.25, *t* = −5.03, *p* < 0.01
	Gr2	*β* = 0.42, *SE* = 0.19, *t* = 2.28, *p* = 0.02
	Target: Gr1	*β* = 1.52, *SE* = 0.32, *t* = 4.78, *p* < 0.01
Tukey contrasts on GJs	Gr1 vs. Gr2	*β* = −1.70, *SE* = 0.27, *t* = −6.40, *p* < 0.01
	Gr1 vs. Gr3	*β* = −1.28, *SE* = 0.25, *t* = −5.03, *p* < 0.01
	Gr2 vs. Gr3	*β* = 0.42, *SE* = 0.19, *t* = 2.28, *p* = 0.02

Both Group 1 and Group 2 read significantly faster than Group 3. Genitive case was significantly different from dative, while locative was not. Out of four interactions (Group 1 by genitive, Group 2 by genitive, Group 1 by locative, and Group 2 by locative), only the first was significant. We also used multiple comparisons (Tukey’s contrasts) to estimate pairwise differences among the three groups and three cases.^7^ Group 1 was significantly faster than the two other groups, and Group 3 was significantly slower than Group 2. Genitive forms took significantly longer to process than both locative and dative, while the difference between the latter two was not significant.

The differences among the three groups were presumably partly due to their proficiency level (Group 1 was the fastest). As for the two beginner groups, Group 2 read faster than Group 3, but made more GJ errors, as we will show below. The differences between case forms let us conclude that despite different proficiency levels, native languages, and experimental settings, all groups exhibited the same non-native-like pattern of online results. As for the significant ‘Group 1 by genitive case’ interaction, it reflects the fact that this non-native-like difference between genitive forms vs. locative and dative forms was less pronounced in the more proficient Group 1 than in the two beginner groups.

##### Grammaticality Judgments

Analyzing the three groups separately, we found that Group 1 demonstrated a native-like difference between target and control errors, while the two beginner groups did not. To estimate this difference statistically, we took all judgment data for ungrammatical sentences from the locative and genitive sets (containing target and control errors) and ran a mixed-effects logistic regression. The factors of interest were the error type (target vs. control) and the group. Control errors and Group 3 were taken as reference levels. For all statistically significant results, model outputs are given in Table 5.

Both Group 1 and Group 2 were significantly different from Group 3. The error type factor was not significant. The ‘Group 1 by target error’ interaction reached significance, while the ‘Group 2 by target error’ interaction did not. In addition, multiple comparisons (Tukey’s contrasts) showed significant differences among all three groups.

The upper-intermediate Group 1 made the fewest errors, and, as we already noted above, the beginner Group 3 made fewer errors than the beginner Group 2, but read more slowly. The interactions show that Group 1 treated target errors differently than Group 3 (namely, they were more difficult to judge than control errors, as they are for native speakers), while Group 2 did not differ from Group 3. We can conclude that the upper-intermediate Group 1 developed a native-like sensitivity to grammaticality illusions that is absent in the beginner groups. However, this sensitivity is evident only in offline, but not in online measures.

## General Discussion

The central question in the field of L2 processing is whether mechanisms and strategies are the same for L1 and L2. In the introduction, we presented different approaches arguing for opposite answers to this question. We suggested that this question may be addressed by focusing on processing problems characteristic of native speakers. If L2 learners attain native-like processing efficiency at a certain proficiency level, they may do so by relying on non-native-like mechanisms and strategies. Developing native-like problems is definitely not the goal of the acquisition process – they are likely to be a by-product of using the same mechanisms as those of the native speakers.

We turned to grammaticality illusions as a well-studied type of processing problem. Slioussar and Cherepovskaia (2014, 2021) demonstrated that the native speakers of Russian were likely to miss particular case errors in the context of a morphologically ambiguous adjective. This was evident in word-by-word RTs and in GJs, both in online and offline measures. The experiment we conducted demonstrated that at the beginner level, L2 readers differed from native speakers in online and offline measures. The online pattern will be discussed below, while offline, there were no significant differences across conditions; this is exactly what we expect in the absence of grammaticality illusions. At the upper-intermediate level, the online pattern remained the same, but a native-like pattern emerged in GJs. We interpret this as evidence in favor of similar processing mechanisms that L2 learners can rely on once the mental representation of nominal inflection develops to a certain extent.

As for the differences between online and offline measures, all models postulating the same processing mechanisms for L1 and L2 recognize that L2 processing is cognitively more demanding, due to lower automaticity and speed, the limitations in lexical access, etc. Several previous studies demonstrated that L2 learners perform better in offline tasks than in online ones (e.g., Hopp, 2010; López Prego and Gabriele, 2014). In these studies, “better” meant “more native-like.” In the present study, we show that L2 learners are more native-like offline even when this does not mean better performance – i.e., when being more native-like means being susceptible to grammaticality illusions.

Now let us turn to online measures, starting with a general picture. Many studies have found differences between different case forms presented in isolation in a variety of languages, including Russian (e.g., Lukatela et al., 1978; Niemi et al., 1991; Gor et al., 2017, 2019; Vasilyeva, 2018). These differences could be explained by the type frequency (even when the token frequency was controlled for) and by syncretism. Gor et al. (2017, 2019), who compared L1 and L2 speakers of Russian, discovered that some distinctions found for native speakers are not (always) observed for L2 learners. In particular, all participants processed nominative forms faster than oblique case forms, and native speakers also processed genitive forms faster than instrumental ones (genitive is the most frequent of the oblique cases). L2 learners showed similar differences only at a certain proficiency level and in a certain experimental design specifically drawing attention to inflectional morphology.

Hyönä et al. (2002), working with Finnish, compared form processing in isolation and in a sentential context and found that many distinctions found in the former situation disappear in the latter. Experiments on Russian (Slioussar and Cherepovskaia, 2014, 2021; Chernova et al., 2020) confirm this generalization. In a sentential context, only sentence-level factors played a role: grammaticality and factors like grammaticality illusions. In particular, in the absence of grammaticality illusions, different ungrammatical forms were processed equally slowly, independently of their case frequency and other properties.

Non-native speakers demonstrate the opposite pattern. While the previous studies showed that they are *less sensitive* to different characteristics of case forms in isolation than L1 speakers are, our study demonstrates that they are *more sensitive* to these characteristics in a sentential context. We hypothesize that native speakers retrieve some form characteristics automatically (hence the effects in isolation), but, when parsing a sentence, they can predict a particular case, which makes these characteristics irrelevant. Non-native speakers are less effective at both tasks, which produce the mirror picture.

In our study, we compared genitive, dative, and locative plural forms and found that both beginner and upper-intermediate L2 learners processed genitive forms significantly slower than locative and dative ones. In a study comparing different oblique case forms in isolation (Vasilyeva, 2018), genitive and accusative forms produced the shortest reaction times, because these cases are much more frequent than other oblique cases.^3^ This factor did not play a role for our L2 participants. As for the order of acquisition, L2 learners of Russian acquire genitive after locative, but before dative (e.g., Rubinstein, 1995a,b; Cherepovskaia et al., 2021).

As far as we can judge, the only factor that can explain this pattern is morphological complexity: how many affixes are associated with a particular form and how complex the rules are that regulate the choice among them. Locative and dative plural have one affix each, with two different orthographic variants depending on the last consonant of the stem. Genitive plural has four affixes with different orthographic variants, and the choice between them depends not only on the last consonant of the stem, but also on the inflectional class and subclass and some other factors. This factor was never found to play a role in L1 processing studies – native speakers use these rules very efficiently.^8^ It would be very interesting to find out whether other properties of noun forms (including case frequency or the order of acquisition) may influence online L2 processing patterns, depending on the experimental design (the task, materials, etc.). But, since the current study is the first processing study comparing different case forms in a sentential context for L2 Russian, further experiments are necessary to answer these questions.

## Data Availability Statement

The raw data supporting the conclusions of this article will be made available by the authors, without undue reservation.

## Ethics Statement

Ethical review and approval were not required for the study on human participants in accordance with the local legislation and institutional requirements. The patients/participants provided their written informed consent to participate in this study.

## Author Contributions

NS came up with the general idea of the study and supervised it at all stages. ER conducted the experiment with the upper-intermediate learners of Russian. NC conducted the experiment with two beginner groups. NC and NS wrote the paper. All authors contributed to the article and approved the submitted version.

### Conflict of Interest

The authors declare that the research was conducted in the absence of any commercial or financial relationships that could be construed as a potential conflict of interest.

## Footnotes

^1^For all inanimate nouns in plural, accusative forms coincide with nominative, while instrumental forms are one letter longer than all other forms, so dative is the optimal choice.

^2^www.neurobs.com

^3^Collecting online and offline data separately would be more challenging for two reasons. Firstly, it is difficult to find two perfectly comparable groups of L2 learners. Secondly, our pilot experiment with more filler sentences and comprehension questions turned out to be difficult even for upper-intermediate participants, so recruiting less proficient participants would be problematic.

^4^www.r-project.org

^5^Slioussar and Samojlova (2015) provide the following counts based on the Russian National Corpus (www.ruscorpora.ru): 30% nominative forms, 26% genitive forms, 5% dative forms, 19% accusative forms, 9% instrumental forms, and 10% locative forms. Other frequency counts based on different corpus samples can be found in Kopotev (2008), but the order of oblique cases remains the same.

^6^Unlike with RTs, model outputs are not presented in a separate table because there are much fewer comparisons.

^7^Since we are looking at two factors with three levels and their interactions, running a mixed-effects regression with a different reference level, as above, was not an option here.

^8^Native speakers also acquire these rules very early and without major problems, while for L2 learners’ morphological complexity is one of the crucial factors that influence case acquisition (Rubinstein, 1995a,b; Cherepovskaia et al., 2021).
